# Nanostructured Tip-Shaped Biosensors: Application of Six Sigma Approach for Enhanced Manufacturing

**DOI:** 10.3390/s17010017

**Published:** 2016-12-23

**Authors:** Seong-Joong Kahng, Jong-Hoon Kim, Jae-Hyun Chung

**Affiliations:** 1 Department of Mechanical Engineering, University of Washington, Seattle, WA 98195, USA; sjkahng@uw.edu (S.-J.K.); jae71@uw.edu (J.-H.C.); 2 School of Engineering and Computer Science, Washington State University, Vancouver, WA 98686, USA

**Keywords:** nanotip, biosensor, sensitivity, specificity, nanofabrication, six-sigma approach

## Abstract

Nanostructured tip-shaped biosensors have drawn attention for biomolecule detection as they are promising for highly sensitive and specific detection of a target analyte. Using a nanostructured tip, the sensitivity is increased to identify individual molecules because of the high aspect ratio structure. Various detection methods, such as electrochemistry, fluorescence microcopy, and Raman spectroscopy, have been attempted to enhance the sensitivity and the specificity. Due to the confined path of electrons, electrochemical measurement using a nanotip enables the detection of single molecules. When an electric field is combined with capillary action and fluid flow, target molecules can be effectively concentrated onto a nanotip surface for detection. To enhance the concentration efficacy, a dendritic nanotip rather than a single tip could be used to detect target analytes, such as nanoparticles, cells, and DNA. However, reproducible fabrication with relation to specific detection remains a challenge due to the instability of a manufacturing method, resulting in inconsistent shape. In this paper, nanostructured biosensors are reviewed with our experimental results using dendritic nanotips for sequence specific detection of DNA. By the aid of the Six Sigma approach, the fabrication yield of dendritic nanotips increases from 20.0% to 86.6%. Using the nanotips, DNA is concentrated and detected in a sequence specific way with the detection limit equivalent to 1000 CFU/mL. The pros and cons of a nanotip biosensor are evaluated in conjunction with future prospects.

## 1. Introduction

Nanomaterials have demonstrated significant impacts in various fields of optics, electronics, composites, energy and sensors. Such impacts are originated from the molecular dimension, which induces relatively large surface area and enhanced interaction with environment. In recent years, various DNA sensors [1,2,3,4,5] have been reported using nanomaterials as an alternative to conventional methods including polymerase chain reaction (PCR)-based detection. Such nanostructured DNA sensors are advantageous not only for their low cost, simplicity and sensitivity, but also for their amenability to miniaturization. Nanomaterials are being used for either the bio-recognition element or the transducer of biosensors to improve the electrochemical signals of biocatalytic event that occur at the electrode/electrolyte interface. In particular, one-dimensional nanostructure generates anisotropic features that render fabrication, operation, and characteristics unique. In the field of biosensors, a nanotip composed of the one-dimensional features offers high sensitivity and specificity due to the isolated targets on nanostructures surface, which can be detected by various mechanisms.

With respect to the fabrication, two kinds of approaches, so called top down and bottom up, have been developed to fabricate a nanostructured tip shape. A top down approach is to etch a bulk material into one-dimensional shape while a bottom up approach is to assemble nanostructures to fabricate a desired shape. To date, top down approaches can offer more uniform shape of nanostructures because well-developed silicon-machining methods are used to precisely engineered nanostructures. However, the scalability and the cost can be limited due to the process compatibility and the high-cost infrastructure. The aspect ratio, i.e., the ratio of length to diameter, for a top down method is smaller than that of a bottom up method. A scanning probe microscope (SPM) tip is a representative product for top down fabrication. The tip diameter and length can be precisely controlled with a resolution of a few nanometers. Using a top down method, nanotips can be fabricated in an array format on a silicon wafer, which shows manufacturing scalability and potential for low cost.

Bottom up approaches are divided into two groups of direct synthesis and assembly after synthesis. Direct synthesis is to grow or synthesize nanostructures on substrate or in liquid using catalyst. Since the sensor geometry is highly dependent on the growth conditions including high temperature, the flexibility to modify the sensor geometry is low. In addition, the stoichiometry is not perfectly controlled to fabricate uniform tip shape, which will directly affect the sensor performance. The assembly after synthesis is popular because nanostructures can be assembled directly on sensor surface with a controlled number, which simplifies the fabrication step and can be scalable. Considering the nature of the assembly in liquid phase, the control over capillary action and other forces during the process is critical to obtain uniform shape of nanotips. Toward a low cost biosensor, the fabrication methods need to be in a massive production fashion and more reliable with standardization.

This paper consists of two parts: (1) a review of nanotip-shaped biosensors with the pros and cons in the contexts of the working principles and the fabrication methods; and (2) our Six Sigma based approach to improve the manufacturing yield for a nanotip sensor. In comparison to the well characterized working principles, the actual development and commercialization is lagged by less reliable manufacturing with lack of scalability. In this paper, the Six Sigma based approach is attempted to reduce the variations and to enhance the scalability and the reproducibility. Using the fabricated structure, amplification-free detection capability for target DNA is demonstrated. The experimental results are discussed with the potential challenges and future directions.

## 2. Nanostructured Tip Bio-Sensors

### 2.1. Fabrication and Nanotip Shapes

**Figure 1:**
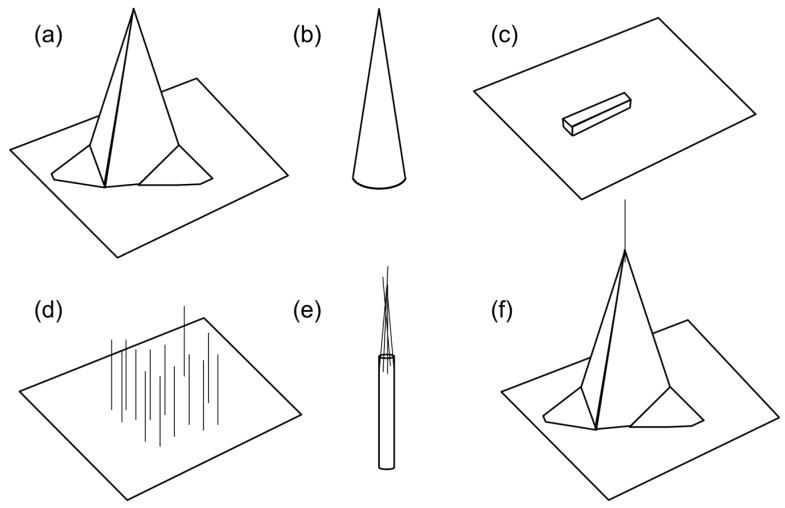
Nanotip geometry based on the fabrication methods: (**a**) conventional atomic force microscope (AFM) tips; (**b**) cone-shaped nanotip; (**c**) nanotip on a flat substrate; (**d**) nanotips grown on substrate; (**e**) nanotips fabricated by capillary action and an electric field; and (**f**) nanotip composed of an AFM tip attached with a nanowire or a nanotube. Panels (**a**–**c**) are fabricated by top down methods; panels (**d**,**e**) are fabricated by bottom-up methods; and panel (**f**) is fabricated by a hybrid method combining bottom-up and top-down methods.

Nanostructured tip biosensors have various shapes depending on the fabrication methods and the application purposes. Figure 1 shows conventional nanotip shape fabricated by top down (Figure 1a–c) and bottom up methods (Figure 1d,e). Figure 1f shows a hybrid method combining top down and bottom up methods. For top down methods, nanotips have been fabricated for an atomic force microscope (AFM) by micromachining process (Figure 1a). The microfarbrication process is well established with batch process to produce nanotips as a well-organized array. The resolution of a nanotip diameter reaches to a few nanometers. Due to the well-defined geometry, the fabricated nanotips have been used for rigorous study of a working principle on a nanotip surface [6,7,8]. A sharp nanotip can also be fabricated by pulling a tungsten wire in liquid or isotropic etching (Figure 1b) [9,10,11]. Nanofabrication process is employed to fabricate a sharp nanotip on planar surface (Figure 1c) [12,13]. However, the nanostructure is laid on surface, which limits the functionalization of the sensing element and the accessibility from biomolecules. In comparison to a suspended nanotip, the nanotip on surface is significantly affected by capillary action. As a result, uniform functionalization of a nanotip surface with polymer and probe molecules is limited. In addition, the nanotip on surface is electrically and optically interfered by substrate in contact.

For bottom up methods, chemical vapor deposition methods can grow nanowires or nanotubes (Figure 1d) [14]. The nanostructures as synthesized can be directly used as a biosensor [15,16,17]. However, the number, the density, and the location of the nanostructures are not precisely controlled, which potentially limits the applicability and the reproducibility. Synthesized nanostructures can be assembled by various methods, such as electric field induced forces, capillary action, electrostatic force (Figure 1e) [18,19,20]. High aspect ratio of nanotips can be fabricated but the tip shape is not uniform and the massive production has not been demonstrated [11]. Nanostructures can be also directly synthesized on an AFM tip surface (Figure 1f) [20,21]. However, the process is cumbersome and challenged for low-cost, scalable fabrication.

### 2.2. Nanotip-Based Biosensors and Potential Challenges

**Figure 2:**
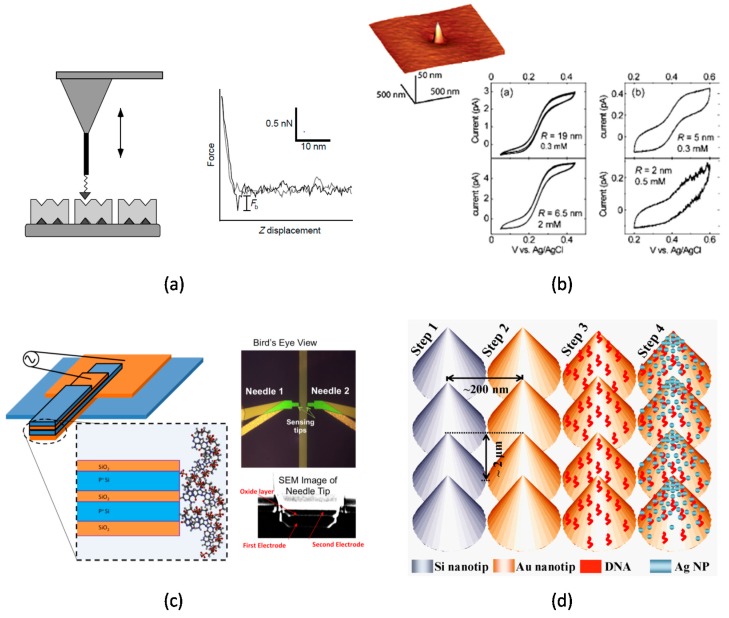
(**a**) (Left) Diagram illustrating a nanotube tip covalently modified with a biotin ligand (dark-grey triangle) interacting with streptavidin protein receptors (light-grey blocks). (Right) Representative force–displacement curve recorded with a biotin-modified nanotube tip on the streptavidin surface in pH 7.0 PBS. The inset represents the scale bar. The binding force is indicated by F_b_. Reproduced with permission from reference [31]. Copyright 1998 Nature; (**b**) (Left) AFM topography image of a nanoelectrode. (right) Cyclic voltammograms obtained for different electrodes recorded in solutions of Fc(CH_2_OH)_2_ and FcTMA. Reproduced with permission from reference [25]. Copyright 2006 American Chemical Society; (**c**) (Left) Schematic of nanoneedle biosensor three-dimensional and side view of horizontal nanoneedles (Not drawn to Scale). (Top right) Optical micrograph of bird’s eye view of aluminum-polysilicon hybrid nanoneedle biosensor. (Bottom right) SEM image of the tip of a nanoneedle biosensor. Reproduced with permission from reference [26]. Copyright 2013 American Institute of Physics; (**d**) A schematic of the pathway for generating the SERS platform for efficient Raman signal enhancement. Reproduced with permission from reference [14]. Copyright 2011 Elsevier B.V.

AFM tips are frequently employed to study target molecules and detection tools for biomolecules [22]. The confined geometry in conjunction with relatively straightforward functionalization protocols facilitates straightforward detection of target molecules [22,23]. When a carbon nanotube (CNT) was attached onto an AFM tip, biological processes and biophysical properties of cells could be monitored with high precision (Figure 2a) [24].

A nanosized electrode fabricated through a nanopore could also confine the geometry of electrodes (Figure 2b) [25]. A nanoneedle fabricated with multiple film layers could be used to detect nucleic acids and proteins (Figure 2c) [26]. A nanosized thin film was fabricated between two insulating layers. After binding of oligonucleotides on the tip region, target DNA was detected by impedance change due to the binding [27]. Using the platform, the specificity was high enough to detect a single mismatch due to the confined geometry. A nanotip was fabricated by etching an optical fiber in liquid. With immobilization of molecular beacons (MBs) at the fiber end, DNA was detected [28].

A CNT based electrode could be fabricated by using electrostatic force [29], which was useful for electrochemical detection due to the confined geometry [30]. Once a CNT was attached, the nanotip could be functionalized for specific detection [31]. When a multi walled carbon nanotube (MWCNT) was attached, the sensitivity for potentiostatic amperometry was significantly improved to detect dopamine in comparison to a tungsten tip without CNTs [32]. A CNT assembled on a tungsten probe could be used to immobilize a few proteins, which can be useful for proteomics due to the designated target [33].

Using nanotips, various biosensors could be fabricated to detect biomolecules. A NiCo_2_O_4_ nanoneedle was fabricated by a liquid-phase chemical growth method [34]. When glucose oxidase was immobilized on the nanoneedle, voltage signal was detected and correlated with glucose concentrations. CNTs grown by chemical vapor deposition were also successful to detect glucose with enzyme reaction [35].

A nanotip structure was advantageous for Raman spectroscopy. A silicon nanotip was fabricated by a chemical vapor deposition method, which was coated with a gold layer for DNA concentration and detection (Figure 2d) [14]. Raman scattering efficiency was improved by using the nanotip coated with a gold layer and silver nanoparticles. Such tip-enhanced Raman spectroscopy (TER) could enhance the sensitivity because of the enhanced resonance on a sharp tip [6]. Using a nanoprobe, the electromagnetic field significantly increased to enhance the sensitivity for surface-enhanced Raman spectroscopy (SERS) [36].

Considering the prior arts, it was clearly found that the confined geometry of a nanotip could enhance the sensitivity of a biosensor because the size of a sensing element was similar to that of a molecular analyte. More importantly for biosensors, established functionalization protocols could be directly applied for specific detection [31]. In spite of the great potential, remaining challenges for commercial-level biosensors are scalable and reliable fabrication, and enhanced sensitivity and specificity. Scalable and reproducible manufacturing of a nanotip is a crucial challenge to achieve a high performance and low cost biosensor. A bottom up method using synthesized nanomaterials can potentially offer an inexpensive solution. However, the challenge for uniform and scalable fabrication should be clearly addressed to success.

In terms of sensitivity, although the sensitivity of a nanotip structure is high enough to detect individual target molecules, a target analyte needs to be bound onto the sensor surface for detection. When the number of target analytes is very small (e.g., 10 molecules/mL), significantly long time is required to bind target to nanotip surface. Owing to the molecular geometry, diffusion limited process requires long incubation time to bind a target to a nanotip due to a governing effect of Brownian motion in molecular dimension [37,38]. In case of a high concentration of target analyte, such as glucose, the sensitivity is not a concern. However, when the concentration becomes low, a concentration mechanism is needed to attract a target onto a nanotip surface.

In our previous work, an electric field was applied to a nanotip to concentrate a target analyte on a nanotip surface. Since the electric field was increased over 10^7^ V/m on a nanotip surface [38], the concentration efficacy could be significantly increased [37,38,39]. A dendritic structure composed of a large number of nanotips could cover larger volume of liquid and increase the sensitivity because various points of a high electric field were generated by the dendritic nanotips [37]. Capillary action could enhance the sensitivity because the compressive pressure at the meniscus around a nanotip could result in the concentration and capture effects of target analyte in conjunction with viscosity [40,41]. Although the capture using capillary action is nonspecific, size selective sorting could be obtained on a nanotip surface because of the capillary force near the nanotip [40,42]. Using the selective sorting, a target could be collected in the mixture of different size particles through the withdrawal action of a nanotip. The captured DNA using a nanotip could be amplified by real time polymerase chain reaction (qPCR) without further purification [42]. The nanotip composed of CNTs and SiC nanowires was dissolved in a PCR tube, which allowed for direct amplification of DNA in the reactor. The concentrated target on a nanotip could be detected by measuring electric current change. Due to the low redox potential of a CNT tip in deionized water, the binding of target bacteria could be sensitively detected [43]. However, the high electric field could locally increase the temperature on a nanotip surface and potentially cause electrical breakdown of electrolyte or target analyte, which can potentially decrease the specificity of a biosensor. A specific detection should be further addressed in case of an electric field induced concentration.

In summary, a nanotip can be a powerful tool for biosensor due to the high aspect ratio. Electric fields could be used to effectively concentrate target analytes. The confined geometry of a nanotip limited the electric current through a terminal end of a tip, which could increase the sensitivity. Raman spectroscopy, fluorescence microscopy, and atomic force microscopy clearly demonstrated the efficiency of the detection due to the molecular geometry. The capillary action on a nanotip surface could be utilized for sorting of target molecules according to the size. However, the challenges of scalable fabrication, specificity and sensitivity still need to be addressed for high performance and inexpensive biosensors. In the following sections, we introduce an engineering approach based on the Six Sigma to address the manufacturing challenge of a nanotip.

### 2.3. Six Sigma Approach and Its Application to Nanomanufacturing

**Figure 3:**
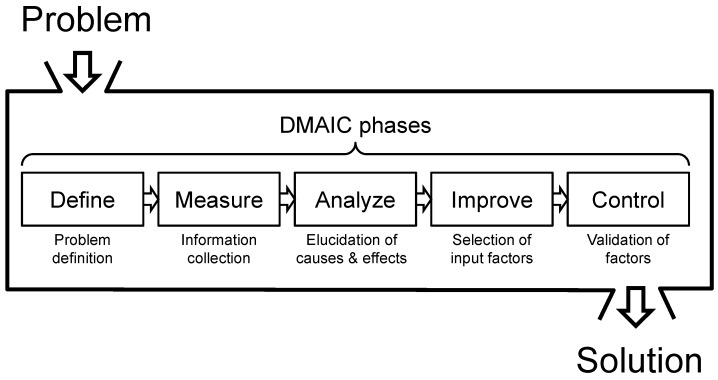
DMAIC phases of the Six Sigma approach; DMAIC represents Define, Measure, Analyze, Improve, and Control. This approach is adapted to improve the quality and the yield for nanotip fabrication process.

The Six Sigma approach is one of the quality management methods to improve a manufacturing process for higher yield and better quality. The approach is composed of five phases: Define, Measure, Analyze, Improve, and Control (DMAIC) (Figure 3) [44]. The Define phase specifically identifies what a problem is, such as yield and quality. The Measure phase collects the information of current level in the contexts of a target problem. The purpose of the Analyze phase is to elucidate the potential causes for a problem and to select a few dominant causes among a number of inputs. In the Improve phase, the selected input factors are applied to enhance product quality. After characterizing the resulting yield, the final stage of the Control phase manages the validated factors that improve the yield and quality.

With respect to the application of the Six Sigma to the nanomanufacturing of biosensors, various factors should be considered. Based on scientific reasoning and engineering principles, the Six Sigma is to enumerate various potential factors and characterize the selected factors to enhance the yield and quality. According to our previous experience, it is interestingly observed that as the yield increases, the quality typically increases. To date, few reports have been found to apply the Six Sigma for analyzing nanomanufacturing process. For example, synthesis of quantum dots and nanowires and device testing were analyzed on the basis of the Six Sigma approach [45]. However, the resulting yield or other outcomes were not clearly mentioned.

In this paper, the Six Sigma approach will be used to improve the nanotip fabrication process. The yield and the tip geometry will be analyzed on the basis of the improvement. Using the fabricated nanotips, sequence specific detection of DNA will be conducted in the following sections.

## 3. Materials and Methods

### 3.1. Application of the Six Sigma Approach to Nanotip Fabrication

To improve the yield and quality of nanotip, the Six Sigma approach was applied to a nanotip fabrication process. The process was to fabricate nanotips using nanowires suspended in liquid by an electric field and capillary action. The process is described in the fabrication section. This section explains how to apply the Six Sigma to the nanotip fabrication.

- Define phase: The goal is to improve the yield of a dendritic nanotip.
- Measure phase: The production yield of a nanotip is 20%. The shape of a nanotip is not uniform.
- Analyze phase: The electric field for tip fabrication can be low, and the medium for carbon nanotubes can be degraded, which can lower the production yield of a nanotip.
- Improve phase: A higher electric field is applied, and the medium for carbon nanotube suspension is refreshed weekly.
- Control phase: When the factors of an electric field and carbon nanotube medium are controlled, the yield is improved from 20% to 80%. The uniform shape is improved.

In the Define phase, the project goal was to improve the yield of a dendritic nanotip. In the Measure phase, it was found that the yield of a nanotip was 20% with inconsistent shape of a nanotip. In our previous report [40], nanotips could be used to detect nonspecifically bound DNA molecules. However, such nanotips might not be used for sequence-specific detection of DNA because the shape of a nanotip was not uniform. Due to the irregular shape of the nanotips, the background noise was relatively high and the error bars for various concentrations were overlapped. A uniform shape was crucial to identify bound DNA on a nanotip surface in a quantitative way. When the tip fabrication process was repeated to obtain a uniform shape, the production yield was only 20.0% (four successes out of 20 trials).

**Figure 4:**
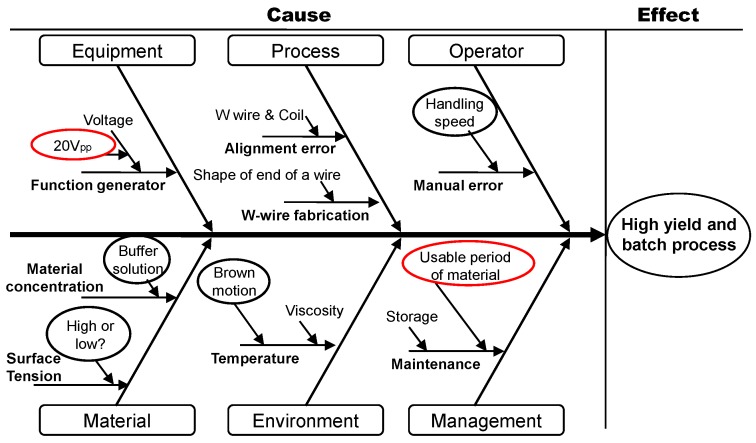
Cause and effect diagram in the Analyze phase for a dendritic nanotip sensor. Equipment, process, operator, material, environment, and management are analyzed to improve yield and scalability. Among them, the red-circled contents of voltage and material usability are investigated in the paper.

The major activity in the Analyze phase was to understand the manufacturing process of the nanotip, including the process flow and to define the potential factors that might affect the low yield and shape inconsistency. Two potential factors of an electric field strength and degradation of N,N-dimethylformamide (DMF) solution suspending nanowires were identified and examined as illustrated in a cause and effect diagram (Figure 4). In investigation of the causes, the fabrication process was segmented into equipment, process, operator, material, environment, and management. In equipment, a function generator (Agilent 33220A, Santa Clara, CA, USA) could generate a maximum peak-to-peak voltage of 20 *V*_pp_, which could be increased to attract a larger number of silicon nanowires in solution. In the process, a homemade setup could generate alignment and fabrication errors. In the operator, the speed of a manually controlled gold-plated tungsten (W)-wire could cause inconsistency. In the material, the concentration of nanowires and the surface tension of solution could cause errors. Temperature change could be a cause for errors in the environment. The solution quality could be changed due to sonication and unidentified chemical reaction in the management. All the potential factors were considered in the Analyze phase.

In the Improve phase, the two activities were selected to enhance the reproducibility on the basis of the Analyze phase: (1) The applied AC voltage was increased from 20 *V*_pp_ to 30 *V*_pp_ for a higher dielectrophoretic (DEP) force because higher voltage could increase the attractive DEP force between W-wire tip and silicon nanowires [37]. The yield was counted among 53 attempts during the 10 days from the prepared date of the silicon nanowire solution. After 10 days, a nanotip was not fabricated, which is described in the result section; (2) A nanotip was fabricated using DMF solution suspending Si nanowires. The characteristic of this solution could affect the fabrication yield and quality. The permittivity of medium was one of the factors for the DEP force [37]. In case of degradation, the permittivity of DMF could be changed to affect the attraction of nanowires in solution. To characterize the effect of DMF on nanotip fabrication, the daily fabrication of nanotips was conducted using the same DMF solution for 14 days. The nanotip shape was compared to characterize the aging effect. In the Control phase, the AC voltage and the usage period (refreshing period) of the DMF solution mixed with silicon nanowire were determined as 30 *V*_pp_ and 7 days, respectively, which will maintain the improved yield and quality.

In summary, two improvements were made according to the Six Sigma approach. One was to increase the voltage from 20 *V*_pp_ to 30 *V*_pp_. The other was to characterize the nanowire solution for two weeks to study the potential degradation of the solution. The details of materials and nanowire fabrication are described in the following sections.

### 3.2. Materials

For a dendritic nanotip fabrication, single-walled carbon nanotubes (SWCNTs) were supplied by Unidym^TM^ (HiPco^®^ SWCNTs, Sunnyvale, CA, USA) and Si nanowires were manufactured in the Nanofab at University of Washington (Seattle, WA, USA). A gold-plated W-wire, 50 µm in diameter, and a silver-plated metal wire were acquired from Sylvania (Towanda, PA, USA) and OK industries (Tuckahoe, NY, USA), respectively. DMF and streptavidin were purchased from Sigma Aldrich (St. Louis, MO, USA). A locked nucleic acid (LNA) was used as a probe molecule, which was supplied from Exiqon (Vedbaek, Denmark). The sequence of a biotinylated LNA probe for the detection of rpoB gene of *Mycobacterium tuberculosis* was 5BioTEG/+GACTG +T +C +GGCGC +T/3BioTEG, where LNA nucleotides are indicated with + in front of the base symbol. The intercalating dye was purchased from ThermoFisher Scientific (DAPI, solution in DMS, Invitrogen, Carlsbad, CA, USA). To evaluate a sensitivity and specificity, *Mycobacterium tuberculosis* (MTB) strain H37Rv, *Mycobacterium avium* complex (*M. avium*), and *Staphylococcus epidermidis* (*S. epidermidis*) were prepared by Cangelosi’s group (Department of Environment and Occupational Health Sciences, University of Washington) with the concentration of 10^6^ CFU/mL. Among the bacteria, MTB was a target analyte. *M. avium* was chosen to study the specificity for a single mismatch case. *S. epidermidis* was chosen to study the specificity for a mismatched case.

### 3.3. Nanotip Fabrication

A dendritic nanotip was fabricated by the assembly of Si nanowires on a gold-plated W-wire followed by the coating of SWCNTs. To achieve the uniform shape of dendritic nanotips, we prepared microfabricated Si nanowires [43]. The conventional photolithography and deep reactive ion etching (DRIE) techniques were used to fabricate Si nanowires on 100 mm Si wafer. The processed wafer was then immersed in DMF and sonicated to collect Si nanowires. SWCNTs were dispersed in DMF by sonication for 10 h. The concentrations of Si nanowire and SWCNT solution were 1 g/L and 100 mg/L, respectively.

**Figure 5:**
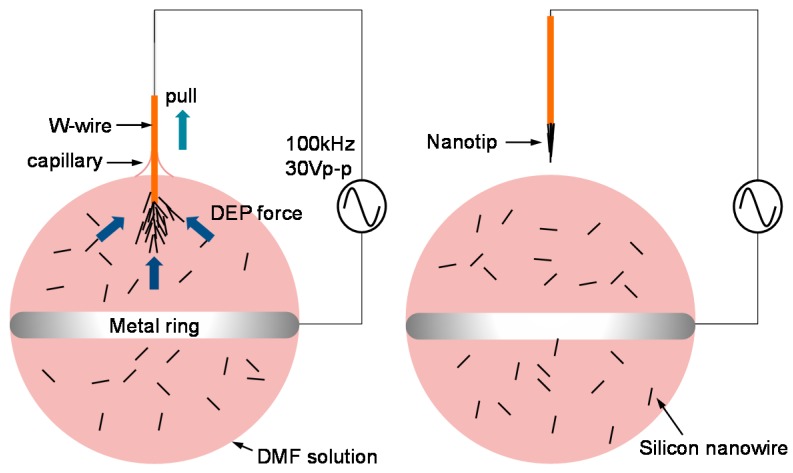
Nanotip fabrication method using an electric field and capillary action; Si nanowires are suspended in DMF. The suspension solution is hung on a metal coil. When a W-wire is withdrawn from the solution, a nanotip composed of Si nanowires is fabricated. The nanotip is again immersed in a SWCNT solution drop in order to coat SWCNTs.

For the nanotip fabrication, 2 µL of a Si nanowire solution drop was placed in a ring-shaped electrode made of silver-plated metal coil (Figure 5). A W-wire was then positioned perpendicularly to the ring-shaped electrode and immersed in the drop with an AC potential (30 *V*_pp_, peak-to-peak voltage) at 5 MHz. Note that the voltage was increased to 30 *V*_pp_ according to the Six Sigma approach. The W-wire was manipulated by using an XYZ stage. After 1 min of immersion time for attraction of Si nanowires, the W-wire was gently withdrawn from the drop. Subsequently, the solvent was removed on a hot plate at 300 °C for 30 min.

After the nanotip made of Si nanowire was fabricated, the Si nanotip was immersed in 2 µL of a SWCNT solution drop. With withdrawal of the nanotip from the solution drop, SWCNTs were coated onto the surface by capillary action and viscosity. Finally, a thin layer of polydimethylsiloxane (PDMS) diluted 10:1 (weight/weight) in Hexane was coated on the nanotip to enhance the adhesion of the nanomaterials. The fabricated nanotip had a conical shape with a dendritic structure because Si nanowires provided the frame of the dendrites. The SWCNTs wrapping Si nanowries offered electrical conduction and adhesion among the nanowires. The average diameter of the terminal end of a nanotip was 0.7–1.2 µm. Based on the same procedure and using the same solution, the nanotip was fabricated for two weeks. The tip shape was compared in order to examine the degradation effect of DMF on the nanotip shape in accordance with the Six Sigma approach.

### 3.4. Nanotip Funtionalization

**Figure 6:**
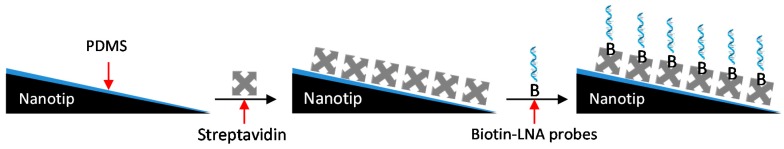
Surface functionalization procedure of nanotip. The nanotip is modified by immersion into a solution containing 1 mg/mL streptavidin for 2 min. The streptavidin coated nanotip is then incubated with biotinylated LNA probe for 5 min.

The nanotips having a uniform shape were used to bind probe molecules. To immobilize the LNA probes, a PDMS-coated nanotip was first dipped in streptavidin diluted with 1× PBS (1 mg/mL) for 2 min for nonspecific protein adsorption. After withdrawing, the nanotip was dried in air for 5 min. Subsequently, it was immersed into 5 µL biotinylated LNA probe for another 5 min (Figure 6). The functionalized nanotip was then moved onto a home-made prototype device for enrichment and hybridization.

### 3.5. Sequence Specific DNA Detection

**Figure 7:**
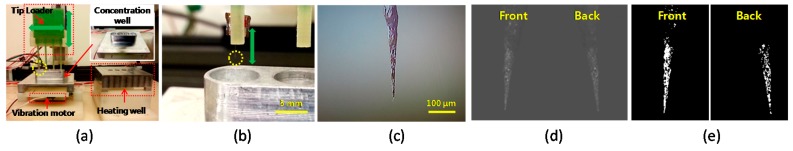
(**a**) Device for DNA concentration with a nanotip; (**b**) a nanotip installed on a coupon is immersed into a concentration well (Magnified image of the yellow circle in Figure 6a; (**c**) optical microscopic image of a dendritic nanotip (magnified image of the yellow circle in Figure 6b); (**d**) fluorescence images of a DNA-captured nanotip; and (**e**) digitized image of the fluorescence images in Figure 6c using a threshold value.

Figure 7a shows the nanotip system designed for the amplification-free and sequence-specific detection of DNA. The system consisted of three major parts: tip loader, DNA concentration well, and heating well. Tip loader included an electrode connected with a function generator to apply an AC electric potential to a nanotip. The tip loader was controlled in x-y directions by two linear motors. X-directional motion was to move a nanotip between a concentration well and a heating well. Y-directional motion was to immerse and withdraw a nanotip in Figure 7b. Figure 7c shows the optical image of a dendritic nanotip. A concentration well that could hold 100 μL solution was installed on a vibration motor, which excited the well for generation of streaming flow in a sample solution. The concentration well also worked as a counter electrode of nanotip. The heating well was utilized to maintain the temperature of buffer for the stringency control.

Figure 7d shows the original image of a nanotip taken by epi-fluorescence microscope (Olympus BX-41, Olympus America Inc., Melville, NY, USA) after DNA capturing and staining. The two images for a nanotip (front and back sides) are taken for digitization. Using a threshold, the images are digitized into black-and-white for detecting target DNA (Figure 7e). After the digitization, the number of white pixels is counted for signal analysis. The log10 of the pixel number was used for actual signal processing. The threshold value was determined to eliminate negative control signals.

**Figure 8:**
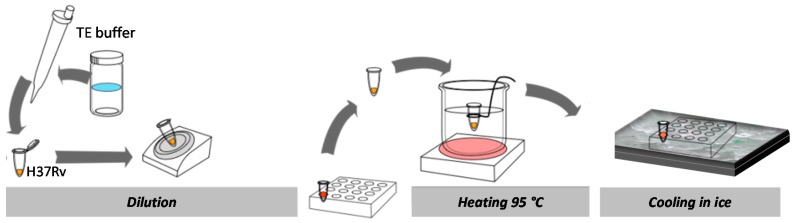
Genomic DNA extraction procedure from cultured cells.

Figure 8 illustrates the extraction procedure for genomic DNA from bacterial cells. The genomic DNA was extracted from bacteria (MTB, *M. avium*, and *S. epidermidis*) with the concentration of 10^4^ CFU/mL by heating at 95 °C for 15 min. The extracted DNA samples were immediately stored in ice to prevent nonspecific binding of denatured DNA until needed. A nanotip shown in Figure 6c was immersed into the concentration well to capture DNA in a sample solution. When the sequence of the attracted DNA was complementary to the LNA probe on a nanotip, DNA was hybridized with LNA probes. Using intercalating dyes (DAPI), the attracted DNA was detected by a fluorescence microscope.

**Figure 9:**
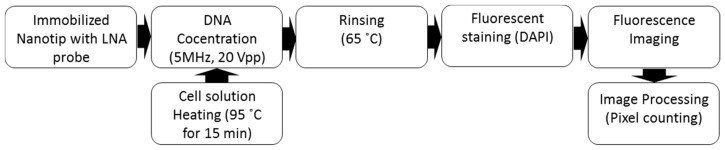
Experimental procedure for DNA concentration and detection using a dendritic nanotip.

Figure 9 shows the experimental procedure for DNA detection with a dendritic nanotip. Using the nanotip immobilized with LNA probes, target DNA molecules were concentrated with the device shown in Figure 7a. DNA was prepared by heating cell solution at 95 °C for 15 min. When a nanotip was dipped in the DNA solution (100 μL), DNA was concentrated with an electric field (5 MHz at 20 *V*_pp_) and streaming flow. Subsequently, non-specifically bound DNA was removed in the 30 s rinsing step in 10 mM Tris buffer at 65 °C. For fluorescent staining, the nanotip binding target DNA was dipped into the solution of intercalating dyes (DAPI) for 5 min. For the fluorescent staining, DAPI of 300 nM was loaded into a well where a nanotip was incubated in DAPI solution. Two fluorescence images were taken on the top and bottom sides of a nanotip surface by epi-fluorescence microscope. Since the captured DNA was shown as a small dot, additional image processing was conducted to digitize the final signal.

For experiment, the optimal concentration time for DNA concentration was studied with 5, 10, and 20 min of concentration times. For this test, rinsing was not conducted in order to study the concentration-time effect on DNA capturing onto nanotip.

To enhance the specificity, the rinsing temperature was varied from 60 °C to 70 °C with the difference of 2–3 °C. MTB (perfectly matched), *M. avium* (single base pair mismatch), and *S. epidermidis* (mismatch) were used for the experiment. After enrichment, it was subsequently washed in heated 1× TE buffer for 15 min for hybridization and stringency control. The hybridization intensity was measured with a fluorescent microscope and analyzed with ImageJ (National Institutes of Health, Bethesda, MD, USA). For verification, 3 experimental runs were conducted for each case.

To test sensitivity and specificity, genomic DNA of MTB cells ranging from 10^2^ CFU/mL to 10^5^ CFU/mL with 10-fold increments was prepared. For the negative control, the functionalized nanotip was immersed into the extraction of *S. epidermidis* (10^4^ CFU/mL) and subjected to the same procedure as above.

## 4. Results

### 4.1. Dendritic Nanotip Fabrication with Six Sigma Approach

**Figure 10:**
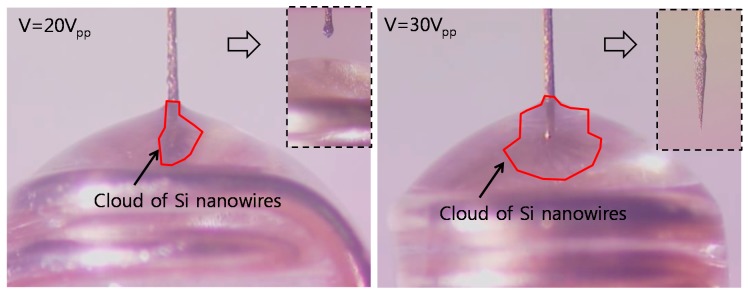
Comparison of nanotip fabrication: 20 *V*_pp_ (**left**); and 30 *V*_pp_ (**right**). The Si nanowire clouds, attracted by the voltages, are expressed by the red lines. The inset shows the fabricated nanotips after withdrawal.

When a nanotip was fabricated, the voltage was increased from 20 *V*_pp_ to 30 *V*_pp_ on the basis of the Six Sigma approach. The increased amplitude of the voltage in the fabrication step increased the dielectrophoretic force, which could attract a larger number of Si nanowires and increased the fabrication yield to 86.6%. Figure 10 compares the nanowires attracted by 20 *V*_pp_ to 30 *V*_pp_. The DEP force enhanced by the increased voltage of 30 *V*_pp_ could form a larger cloud of Si nanowires. The inset image in the left shows a failure case at 20 *V*_pp_ due to the smaller number of Si nanowires while that in the right shows a successful case at 30 *V*_pp_. In the successful case, the attracted nanowires were shaped to a cone due to the compressive force of capillary action.

**Figure 11:**
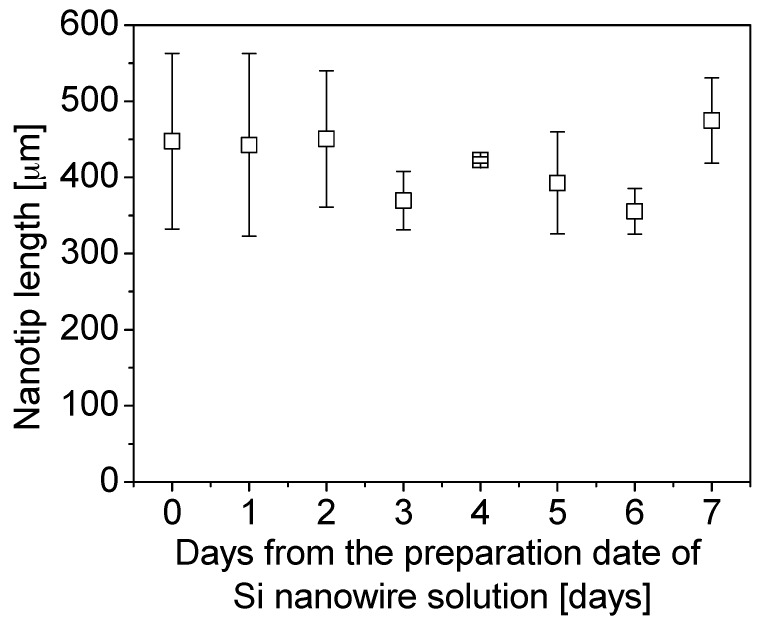
Average lengths of nanotips for 7 days from the preparation date of a Si nanowire solution.

Figure 11 shows the average lengths of nanotip that was measured from Day 0 to Day 7. According to our experimental observation, nanotips were successfully fabricated from Day 0 to Day 10. From Day 11, however, nanotips were not fabricated, possibly due to degradation of DMF solution. With conservative manufacturing decision, nanotips were fabricated for the following DNA experiment by using the Si nanowire solution prepared within 7 days. The average length and the standard deviation of the fabricated nanotips within 7 days were 426.0 ± 94.0 µm for the total 50 nanotips.

**Figure 12:**
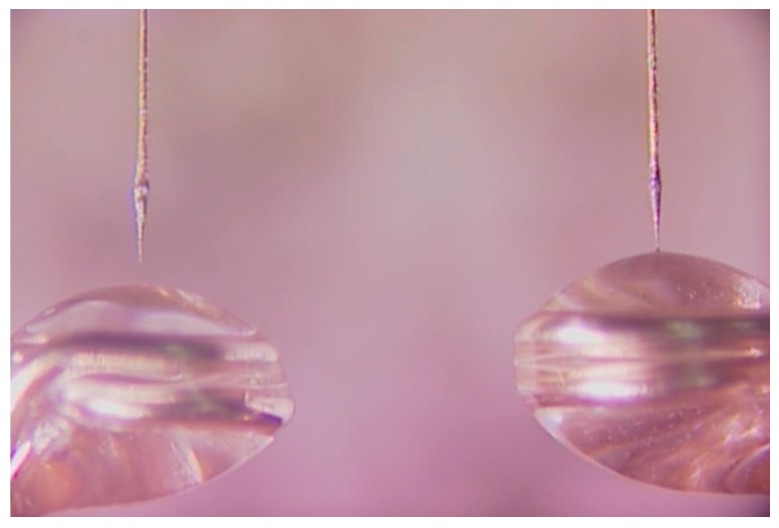
With improving the manufacturing yield of nanotips, two nanotips are simultaneously fabricated using two fabrication setups.

As the manufacturing yield increased, the simultaneous fabrication of two nanotips was attempted to evaluate if an array of nanotips could be fabricated by the same method. As shown in Figure 12, nanotips were simultaneously fabricated from two fabrication setups. In the fabrication, a single voltage source was used because the voltage reduction was not significant in DMF solution. However, two nanotips could not be fabricated in a single drop because the nonsymmetrical shape of the drop interfered with the meniscus near a nanotip. The change of the meniscus could affect reproducible formation of capillary action in the fabrication process. In addition, Si nanowires attracted to both W-wires were tangled in a solution drop, which resulted in the failure of nanotip fabrication.

As results of the Six Sigma application to the nanotip fabrication, it was found that the yield was improved from 20.0% to 86.6%. For conservative manufacturing, the usable period of DMF solution was 7 days. The average and the standard deviation of the fabricated nanotips was 426.0 ± 94.0 µm for 50 nanotips. The nanotips were used for sequence specific detection of DNA as described in the next section.

### 4.2. Sequence Specific DNA Ddetection

**Figure 13:**
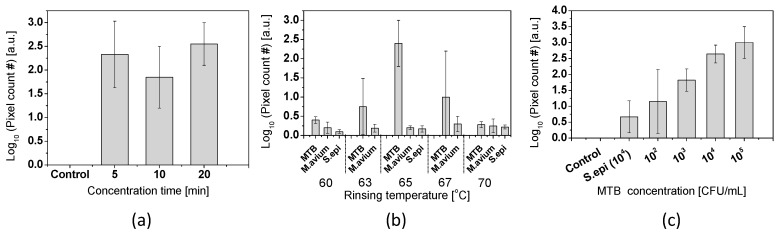
(**a**) Concentration time test (N = 3); (**b**) Specificity result with genomic DNA of MTB, *M. avium*, and *S. epidermidis* at various rinsing temperature (N = 3). The concentration of the bacteria before extraction is 10^4^ CFU/mL. (**c**) Sensitivity test with MTB DNA (N = 3).

To study the optimal immersion time for DNA concentration, tests with 5, 10, and 20 min of concentration times were conducted. As shown in Figure 13a, the average value of pixel counting was similar for 5, 10, and 20 min of concentration times. Based on this result, we chose the concentration time of 5 min. For the rinsing temperature optimization, varied rinsing temperatures from 60 °C to 70 °C with the difference of 2–3 °C were tested with 10^4^ CFU/mL of MTB, *M. avium*, and *S. epidermidis*. The result showed that the highest signal-to-noise ratio can be achieved at 65 °C (Figure 13b), which was used for the sensitivity test. A sensitivity test was conducted with MTB cell solution (Figure 13c). Cell concentration was changed from 10^2^ to 10^5^ CFU/mL by 10-fold increment. As a negative control, pure 1× TE buffer was used instead of MTB cell solution. The sensitivity was 100 CFU/mL. However, in comparison with 10^4^ CFU/mL of *S. epidermidis*, the detection limit was 10^3^ CFU/mL.

## 5. Discussion

According to the Six Sigma approach, the yield of the nanotip fabrication was significantly improved from 20.0% to 86.6%. In comparison to the previous work [40,43], the tip shape became uniform with increase of the yield. The shape of the nanotips was very crucial to control the background noise because the non-uniform geometry of nanotips could increase the nonspecific capture of fluorescent particles, which consequently increased the background noises. It was found that DMF might be degraded according to the time, which reduced the yield to 0% after 10 days. Scientific investigation on DMF degradation related to the prepared solution is needed to validate our results. However, an engineering approach using the Six Sigma could partially address the issue of the low fabrication yield. To further improve the yield and the scalability, all the fabrication and detection procedures need to be investigated on the basis of the Six Sigma approach.

The fabricated nanotips with Six Sigma approach were used to demonstrate sequence specific DNA detection. DNA molecules were attracted onto a nanotip by: (1) circulation flow in the vicinity of a nanotip; and (2) DEP force by an AC electric field. The concentration mechanism was reported in our previous report [46]. For long-range attraction, DNA molecules were delivered by the circulation flow. When the well was vibrated, the surface wave generated the streaming flow to generate the circulation flow. The flow rendered DNA approaching to the vicinity of a nanotip where the DEP force was effective. DEP was the second mechanism of concentrating DNA working in a short range. DEP was generated by the induced dipole of a particle under a non-uniform electric field. The non-uniform electric field from a nanotip generated DEP force to capture DNA onto a nanotip surface. As shown in Figure 13a, 5 min was long enough to concentrate target DNA in the sample solution.

For specific capture of target DNA, LNA probes were utilized to enhance sensitivity and specificity. Unlike conventional oligonucleotide probes, LNA probes have a higher association constant by 10~100 folds [47,48], which allowed us to apply a more rigorous rinsing step for nonspecifically bound DNA. LNA probes were designed to increase the difference of melting temperature between perfectly matched- and non-perfectly matched-DNA to LNA probe. Due to the discrepancy between the designed and the actual melting temperatures, the optimized temperature for rinsing of captured DNA molecules was studied to discriminate DNA molecules with single base pair mismatch from target DNA. The high specificity was achieved at 65 °C (Figure 13b). Note that the hybridization was interfered for both complementary and non-complementary targets at lower temperature shown in Figure 13b, which requires further study to understand the temperature dependence of LNA probe. Thus, more rigorous control of hybridization stringency can minimize the nonspecific interaction and improve the sensitivity and specificity of the nanotip assay.

A nanotip sensing platform has great potential as an inexpensive and sensitive biosensor. Various working principles including optical and fluorescent detection, Raman spectroscopy, and electrochemical measurement can be applied to enhance the sensitivity. In addition to detection, the efficacy of the concentration can be improved by applying an electric field, capillary action, and fluid flow. In consideration of the interdisciplinary nature of the nanotip biosensors, the Six Sigma approach can contribute the improvement of the yield and the quality, which will directly impact the improvement of sensitivity and specificity. Toward amplification-free detection of DNA, more rigorous experimental setup with the refined experimental procedure should be required.

## 6. Conclusions

In summary, a nanotip-based biosensor was reviewed with our experimental results using dendritic nanotips for sequence specific detection of DNA. A dendritic nanotip could be uniformly fabricated by the aid of Six Sigma approach. The yield increases from 20.0% to 86.6%. Genomic DNA was prepared by heating solution containing bacterial cells including MTB strain H37Ra, *M. avium* and *S. epidermidis*. Using the DNA solution of 100-µL-sample volume, DNA was concentrated on a nanotip surface by DEP force and convective flow. The nanotip assay showed the detection limit of 1000 CFU/mL. The single base mismatch (*M. avium*) detection was achieved from the LNA probe immobilized on the nanotip surface. The sequence specific DNA detection could be completed within 30 min without amplification. While the current configuration of the nanotip assay requires fluorescence detection, it can potentially be used with electrical detection due to the uniform structures.
